# The research for PLTS normalization method based on minimum entropy change and its application in MAGDM problem

**DOI:** 10.1371/journal.pone.0268158

**Published:** 2022-05-06

**Authors:** Shouwen Wu, Xianfeng Chen

**Affiliations:** 1 School of Computer and Information Engineering, Henan University of Economics and Law, Zhengzhou, China; 2 Beijing Union University, Beijing, China; Sichuan University, CHINA

## Abstract

In the problem of multiple attributes group decision making (MAGDM), the probabilistic linguistic term sets (PLTSs) is an useful tool which can be more flexible and accurate to express the evaluation information of decision makers (DMs). However, due to the lack of time or knowledge, DMs tend to provide the evaluation information by incomplete PLTSs (InPLTSs) which contain missing information. The process to estimate the missing information of InPLTSs is essential, which is called the normalization of InPLTSs. By analyzing the previous methods, the existing defect is that the original uncertainty information of InPLTS can be hardly retained after normalizing. Moreover, the literature that considers the normalization method from perspective of entropy change is absent. Thus, to overcome the shortcoming and fill the research blank, we propose two optimization models based on minimum entropy change of InPLTSs, which can remain the original uncertainty information of InPLTSs to the greatest extent. Inspired by entropy measure of PLTSs, the novel concepts related to entropy measure of InPLTS are developed. In addition, based on the novel normalization method, a decision model is constructed to solve the MAGDM problem. To verify the feasibility and superiority of the proposed method and model, a case about the selection of five-star scenic spots is given and we conduct to have comparative analysis with other methods.

## 1. Introduction

In real world, the decision problems become complex increasingly [1]. Single person difficultly makes accurate and scientific decision owing to the limited time and the lack of knowledge or experience [2]. In this sense, to achieve the efficient and reasonable decision result, the group decision making (GDM) is propounded [3–5], which has been applied to different fields, e.g., supplier selection [6], medical resource management [7] and the selection of investment brands [8]. As we all know, it may contain many attributes in the decision scenario, e.g., material transportation selection including fee cost, time, security and so on. The situation of decision is called multi-attribute group decision making (MAGDM), which has been also applied broadly to many fields, e.g., green logistics risk assessment [9], the selection of automobiles for customers [10] and portfolio allocation [11]. For the MAGDM problem, three steps are conducted generally [12]: (1) collection of evaluation information, (2) information aggregation, (3) ranking process.

However, in most cases, when facing various alternatives and attributes, experts are not able to give complete and accurate evaluations due to the vagueness and hesitance of human cognition as well as the complexity of issue [13]. To model the fuzzy and hesitant information, Zadeh [14] proposed the fuzzy sets (FSs) in decision making. Subsequently, the concept of hesitant fuzzy sets (HFSs) was introduced by Torra [15], in which experts can use several possible values with corresponding membership degrees to describe their preferences. Actually, when facing the decision problem, people prefer to use linguistic terms to express their preferences intuitively rather than crisp numbers [16, 17]. Thus, Zadeh [18] firstly proposed the concept of linguistic valuables (LVs), which describes the preference information by using linguistic words such as ‘good’, ‘medium’, ‘bad’. Afterward, on the basis of HFSs and LVs, Rodriguez et al. [19] developed the hesitant fuzzy linguistic term sets (HFLTSs) so that decision makers (DMs) could utilize a set of linguistic terms to describe their preferences and it well expresses the hesitance and vagueness of DMs. But the defect of HFLTSs is that the importance or weights of linguistic terms are identical as default. In reality, the importance of every linguistic term provided by DMs is different because of the cognitive complexity of human and the distinct preference degrees over schemes [20]. Moreover, HFLTSs can not be used to describe the evaluation information of group. For example, when evaluating a scheme, 20% of DMs support alternative A and 30% of DMs reject alternative A and 50% of DMs express neutrality. Hence, it can not use HFLTSs to precisely describe this situation with different proportional information. To overcome the shortcomings of HFLTSs, Pang et al. [5] developed the concept of probabilistic linguistic term sets (PLTSs) that the set contains not only various linguistic terms but also the corresponding probabilities. PLTSs can largely retain the ambiguous information of DMs and reflect the importance distribution of LTs. In recent years, PLTSs has become the research focus due to its advantages and potential. For a PLTS, it contains one or more linguistic terms and the sum of probabilities of all linguistic variables may be not equal to 1, i.e., $\sum_{k=1}^{\#L(p)}p^{\left(k\right)}\leq 1$ (where #*L*(*p*) denotes the number of linguistic terms in a PLTS). There are numerous literature involving the PLTSs since the theory was proposed, and some open and potential research problems of PLTSs have been also revealed. Xu et al. [21] gave an overview of probabilistic-based expressions from characteristic, technology, comparison methods, advantage and application. Liao et al. [22] and Mi et al. [23] reviewed the research status of PLTSs from some aspects and provided the existing problem and future directions respectively. Considering that DMs interpret the linguistic information in different ways, Wan et al. [24] proposed a novel expression model based on individual semantics, which takes the cognitive differences and psychological behavior of decision makers into account. On the problems of PLTS operators, Xu et al. [25] considered whether the elements are independent and proposed some novel operators of PLTS, which were used to fuse DMs’ information in group decision making. Wan et al. [26] developed a new possibility degree to rank PLTSs and defined some operational laws on the basis of Archimedean copulas and co-copulas.

Among the researches on PLTSs, the normalization method of PLTSs is an essential issue. Different normalization methods will influence the original preference information provided by DMs and the final decision results. Therefore, how to design a scientific and reasonable normalization method is a meaningful research topic. The main tasks of PLTSs normalization contain two aspects [5]: (1) estimate the unknown probabilities if the sum of linguistic terms probabilities is less than 1, i.e., $\sum_{k=1}^{\#L(p)}p^{\left(k\right)}<1$; (2) unify the number of LTs when comparing or operating between two PLTSs. For the second task above, the common method is to make #*L*_1_(*p*) = #*L*_2_(*p*) (where #*L*_1_(*p*) represents the number of LTs in *L*_1_(*p*)). If #*L*_1_(*p*)<#*L*_2_(*p*), add the minimum elements into *L*_1_(*p*) whose probabilities are set 0 and vice versa. It is not more meaningful to study the second problem so this paper mainly focuses on the first task. For the first task, Pang et al. proposed a normalization method that unknown probabilities are assigned averagely into the known LTs. Pang’s normalization method is most popular due to its simplicity of calculation. Moreover, there are four other approaches to normalize PLTSs [23]: (1) Full-set assignment; (2) Power-set assignment; (3) Envelope assignment; (4) Attitudes assignment. Recently, Wang et al. [27] summarized the incomplete PLTS (InPLTS) as two types, namely, InPLTS with missing LTs (MLT-InPLTS) and InPLTS with missing probabilities (MP-InPLTS), and constructed several optimization models to estimate the missing information based on preference consistency. For the two types of InPLTSs, some scholars proposed many corresponding methods. To solve the problem of MLT-InPLTS, Song [28] and Song et al. [29] proposed a probability computing model to estimate the missing probabilities respectively. Tian et al. [30] developed a personalized normalization method through the two-stage decision-making process, which integrated three types of individual risk attitudes. For the MP-InPLTS, Gao et al. [31, 32] utilized the emergency fault tree analysis (EFTA) to estimate the missing probabilities in emergency decision making. However, there exist many limitations in previous normalization methods, which are shown as follows:

1. Although it is straightforward and simple to use Pang’s method, the defect is that it ignores the situation that the missing probabilities may belong to the unknown LTs. Moreover, it can not be applied in some special cases. Thus, it is not reasonable and scientific to use Pang’s method.
2. For the above approach (1) ~ (3), the process of calculation is very difficult owing to the massive added elements, especially when dealing with the complex decision problem. For approach (4), it only applies to the situation that considers the risk preferences of DMs. Hence, the application area of approach (4) is narrow.
3. Although some estimation models are proposed in [27] to reasonably solve the MLT-InPLTS and MP-InPLTS, the calculations are more cumbersome. It can not be applied to the situation of single InPLTS. In other words, the method can be only applied to some specific scenarios that consider the consistency and group consensus.
4. To the best of our knowledge, the literature of normalization method that considers from the angle of entropy change is absent. In previous studies, it ignores that the original uncertainty information of InPLTS may change after the normalization process. And the change of uncertainty information of InPLTS will influence the final results of decision. Thus, it is necessary to consider the change of uncertainty information when conduct the normalization of InPLTS.

The entropy was originally used to measure the uncertainty of probabilistic information [33]. Afterward, it was extended to express the uncertainty information of fuzzy set [34, 35]. To incorporate the subjectivity in the fuzziness measure, a weighted fuzzy entropy was introduced [36]. Xu et al. [37] introduced the concepts of entropy and cross-entropy for hesitant information and discussed their properties. Subsequently, Xu et al. [38] reviewed the existing entropy measure for hesitant fuzzy elements and adopted the two-tuple model to represent the uncertainty in some special cases. To express the uncertainty of PLTSs, Liu et al. [33] introduced the concepts of fuzzy entropy, hesitant entropy and total entropy of PLTSs. Generally, it contains two kinds of entropy, namely, fuzzy entropy and hesitant entropy. The fuzzy entropy mainly describes the distance distribution between LTs and the middle LT in a LTS, and the hesitant entropy describes the dispersion degree among the LTs in a LTS. Lin et al. [39] proposed the concept of PLTS information entropy and gave its formula, but it only used the probability information of PLTS, which may lead to the inaccuracy of uncertainty information measure. To overcome the shortcoming, Xu et al. [40] proposed some improved entropy measure methods of PLTS from the perspective of expected value including hesitant entropy, fuzzy entropy and cross entropy of PLTS. Entropy is an important tool to measure the uncertainty information of PLTSs. Considering the existing defects of previous normalization methods of InPLTS, one important principle is to remain the original information of PLTS as much as possible. Hence, we develop two optimization models to estimate the missing probabilities based on minimum change of entropy. The proposed method can remain the original uncertainty information of InPLTS to the greatest extent.

The main work and contributions in this paper are as follows:

1. Inspired by previous entropy measure of PLTS, we give some definitions of InPLTS entropy, which includes fuzzy entropy and hesitant entropy of InPLTS. The computing methods of entropy change of InPLTS are proposed on the basis of Liu’s method. Besides, we also define the concepts of entropy change of InPLTS.
2. To estimate the MLT-InPLTS and MP-InPLTS, we construct two optimization models based on minimum change of entropy. The models can cope with any InPLTS, which can retain the original uncertainty information of InPLTS and need not depend on other additional information.
3. We design a novel MAGDM model based on the proposed normalization method of InPLTS, adopting the aggregation operator and expected value of PLTS in the process of information fusion and ranking, respectively. The model can solve the MAGDM problems faster and more accurately.
4. The proposed MAGDM model is used to solve the problem of five-star scenic spot selection, and we make a comparative analysis with other methods.

The remaining contents are arranged as follows. In section 2, there are some basic concepts and definitions including LTSs, PLTSs, InPLTSs, Entropy measure of PLTSs and so on. After that, we give some definitions of InPLTS entropy and propose the normalization methods of InPLTSs based on minimum entropy change in section 3. In section 4, a MAGDM model is constructed to solve the decision problem. A case about the selection of five-star scenic spots is conducted to illustrate the MAGDM model in section 5 and we have a comparative analysis in section 6. Finally, the conclusion is given in section 7.

## 2. Preliminaries

In this subsection, some basic concepts are given including LTSs, PLTSs, InPLTSs and some equations of PLTS entropy measure.

### 2.1 LTS and PLTSs

Zadeh [18] put forward the concept of linguistic valuables(LVs) so that DMs can utilize the linguistic valuables to depict their preferences. LTS is a set consisted of linguistic elements, and its mathematical form as follows:

**Definition 1** [41, 42] Let *S* be a set, *S* = {*s*_*α*_|*α* = 0, 1, 2, …, 2*τ*}, where *τ* is a positive integer and odd, and the element *s*_*α*_ represents the corresponding linguistic variable, such as ‘*s*_0_ = terrible, ‘*s*_1_ = indifference, *s*_2_ = perfect’. Thus, the set *S* is called a LTS, which has some properties below:

1. *s*_*a*_ *>s*_*b*_, if *a* >*b*.
2. Neg(*s*_*α*_) = *s*_2*τ*-*α*_, where Neg is the symbol of negation operator.

To reflect well the distinct distribution of probabilities, Pang et al. [5] proposed the concept of PLTS, which is more flexible and accurate than HFLTS to describe the preferences of DMs.

**Definition 2** [5] Let *S* be a LTS, *S* = {*s*_*α*_|*α* = 0, 1, 2, …, 2*τ*}. Suppose that *L*(*p*) is a PLTS:

$$L(p)=\left\{L^{\left(k\right)}\left(p^{\left(k\right)}\right)|L^{\left(k\right)}\in S,p^{\left(k\right)}\geq 0,k=1,2,\ldots ,\#L(p),\sum_{k=1}^{\#L(p)}p^{\left(k\right)}\leq 1\right\},$$

where *L*^(*k*)^ denotes the *k* th LT of *S* and *p*^(*k*)^ is the probability of *L*^(*k*)^. #*L*(*p*) is the number of LTs in the *L*(*p*). When $\sum_{k=1}^{\#L(p)}p^{\left(k\right)}<1$, the PLTS needs to be converted to the normalized form and the normalized PLTS is noted as NPLTS. The form of NPLTS is given by

$$\dot{L}(p)=\left\{L^{\left(k\right)}\left({\dot{p}}^{\left(k\right)}\right)|k=1,2,\ldots ,\#L(p)\right\},$$

where ${\dot{p}}^{\left(k\right)}=p^{\left(k\right)}/\sum_{k=1}^{\#L(p)}p^{\left(k\right)}$.

**Remark 1** Although the above normalized method seems much simple in the process of calculation, it has evident defects in some cases. The nature of this method is to enlarge all the existing elements in PLTS in equal proportion, which often produces contradiction, e.g., two PLTS *L*_1_(*p*) = {*s*_0_(0.2), *s*_1_ = (0.3)} and *L*_2_(*p*) = {*s*_0_(0.4), *s*_1_(0.6)}. After the normalization process, *L*_1_(*p*) is changed to ${\dot{L}}_{1}(p)=\left\{s_{0}(0.4),s_{1}(0.6)\right\}$, which is equal to *L*_2_(*p*). Obviously, it is unreasonable. Besides, when several PLTSs have only one LT with incomplete probability, e.g., *L*_3_(*p*) = {*s*_0_(0.4)}, *L*_4_(*p*) = {*s*_0_(0.5)}, *L*_5_(*p*) = {*s*_0_(0.6)}. Then, according to the above normalization method, the results are ${\dot{L}}_{3}(p)=\left\{s_{0}(1)\right\}$, ${\dot{L}}_{4}(p)=\left\{s_{0}(1)\right\}$, ${\dot{L}}_{5}(p)=\left\{s_{0}(1)\right\}$. Hence, the normalized results by using the method in literature [5] are not scientific and reasonable.

For the convenience of operating among PLTSs directly, Mao et al. [43] proposed the concept of ascending ordered PLTS as follows:

**Definition 3** Given a PLTS *L*(*p*) = {*L*^(*k*)^(*p*^(*k*)^)|*L*^(*k*)^ ∈ *S*, *p*^(*k*)^ ≥ 0, *k* = 1, 2, …, #*L*(*p*)}, where *r*^(*k*)^ represents the subscript of linguistic term *L*^(*k*)^, and an ascending ordered PLTS can be derived by the following steps:

1. If all values of *r*^(*k*)^*p*^(*k*)^ in a PLTS are different, then all elements are arranged according to the value of *r*^(*k*)^*p*^(*k*)^ in an ascending order;
2. If there are two or more identical values of *r*^(*k*)^*p*^(*k*)^ in a PLTS, then
  - When the subscripts *r*^(*k*)^ are different, *r*^(*k*)^*p*^(*k*)^ are arranged according to values of *r*^(*k*)^ in an ascending order;
  - When the subscripts *r*^(*k*)^ are identical, *r*^(*k*)^*p*^(*k*)^ are arranged according to values of *p*^(*k*)^ in an ascending order.

Moreover, some basic operations of PLTSs are provided in literature [5].

1. $L_{1}(p)⊕L_{2}(p)=\cup_{L_{1}^{\left(k\right)}\in L_{1}(p),L_{2}^{\left(k\right)}\in L_{2}(p)}\left\{p_{1}^{\left(k\right)}L_{1}^{\left(k\right)}⊕p_{2}^{\left(k\right)}L_{2}^{\left(k\right)}\right\}$;
2. $L_{1}(p)⊗L_{2}(p)=\cup_{L_{1}^{\left(k\right)}\in L_{1}(p),L_{2}^{\left(k\right)}\in L_{2}(p)}\left\{{\left(L_{1}^{\left(k\right)}\right)}^{p_{1}^{\left(k\right)}}⊗{\left(L_{2}^{\left(k\right)}\right)}^{p_{2}^{\left(k\right)}}\right\}$;
3. $\lambda L(p)=\cup_{L^{\left(k\right)}\in L(p)}\lambda p^{\left(k\right)}L^{\left(k\right)},\lambda \geq 0$;
4. $L{\left(p\right)}^{\lambda }=\cup_{L^{\left(k\right)}\in L(p)}\left\{{\left(L^{\left(k\right)}\right)}^{\lambda p^{\left(k\right)}}\right\}$.

Because it contains the probabilities in PLTSs, the expected values are often used to compare two PLTSs or rank alternatives and the calculation process is easy. Pang et al. [5] firstly gave the equation of PLTS expected value.

**Definition 4** [5] Let *L*(*p*) be a PLTS, the expected value is given by

$$E(L(p))=\sum_{k=1}^{\#L(p)}I\left(s_{k}\right)p^{\left(k\right)}/\sum_{k=1}^{\#L(p)}p^{\left(k\right)},$$ (1)

where *I*(*s*_*k*_) represents the extraction function which can get the subscript of *s*_*k*_.

To achieve the collective information, we conduct it with the aid of the aggregation technology. The aggregation operator is a simple and frequently-used tool. For the aggregation of PLTS, Pang et al. [5] introduced many aggregation operators but we may only use the probabilistic linguistic weighted averaging operator(PLWA) in this paper.

**Definition 5** [5] Let $L_{i}(p)=\left\{L_{i}^{\left(k\right)}\left(p_{i}^{\left(k\right)}\right)|k=1,2,\ldots ,\#L(p)\right\}(i=1,2,\ldots ,n)$ be *n* PLTSs, then

$$\begin{array}{c} PLWA\left(L_{1}(p),L_{2}(p),\ldots ,L_{n}(p)\right)=w_{1}L_{1}(p)⊕w_{2}L_{2}(p)⊕\ldots ⊕w_{n}L_{n}(p)\\ =\cup_{L_{1}^{\left(k\right)}\in L_{1}(p)}\left\{w_{1}p_{1}^{\left(k\right)}L_{1}^{\left(k\right)}\right\}⊕\cup_{L_{2}^{\left(k\right)}\in L_{2}(p)}\left\{w_{2}p_{2}^{\left(k\right)}L_{2}^{\left(k\right)}\right\}⊕\ldots ⊕\cup_{L_{n}^{\left(k\right)}\in L_{n}(p)}\left\{w_{n}p_{n}^{\left(k\right)}L_{n}^{\left(k\right)}\right\}, \end{array}$$ (2)

where $L_{i}^{\left(k\right)}$ denotes the *k*th linguistic term in *L*_*i*_(*p*), and $p_{i}^{\left(k\right)}$ denotes the probability of $L_{i}^{\left(k\right)}$. The *w*_*i*_ (*i* = 1, 2, …, *n*) is the corresponding weight of *L*_*i*_(*p*). Thus, PLWA is called the probabilistic linguistic weighted averaging operator.

### 2.2 InPLTSs

In the above, we review some basic knowledge about PLTSs. However, when facing the real decision problem, due to the lack of knowledge or time, DMs are not able to provide the completed PLTSs in most cases, which lead to the missing information of occurrence probabilities in PLTSs. To depict this situation, Gao et al. [32] developed the concept of incomplete probabilistic linguistic term sets (InPLTSs).

**Definition 6** [32] Let *S* = {*s*_*α*_|*α* = 0, 1, 2, …, 2*τ*} be a LTS, where *τ* is a positive integer. Then the InPLTS is given by

$$L(x)=\left\{L^{\left(k\right)}\left(x^{\left(k\right)}\right)|L^{\left(k\right)}\in S,x^{\left(k\right)}\geq 0,k=1,2,\ldots ,\#L(x),\sum_{k=1}^{\#L(x)}x^{\left(k\right)}\leq 1\right\},$$

where *L*(*x*) represents an InPLTS with unknown probabilities. *L*^(*k*)^ and *x*^(*k*)^ denote the *k*th LT and its uncertain occurrence probability. And *L*^(*k*)^(*x*^(*k*)^) represents the probabilistic linguistic element(PLE).

**Remark 2** In section Introduction, two kinds of InPLTS are reviewed, namely, MLT-InPLTS and MP-InPLTS [27]. For the MLT-InPLTS, an example is *L*_1_(*p*) = {*s*_0_(0.2), *s*_1_ = (0.3)}, where the sum of occurrence probabilities is less than 1 so we need assign the remaining probabilities to the known or unknown LTs. Pang et al. [5] assign the missing probabilities to the known elements. In reality, the remaining probabilities should belong to all the possible elements in LTSs [27] and in this paper we consider that it only belongs to one unknown LT *s*_*r*_, *s*_*r*_∈*S* where *r* is a discrete value. For the MP-InPLTS, the example is *L*_6_(*p*) = {*s*_0_(0.2), *s*_1_(*x*_1_), *s*_2_(*x*_2_)}, where the occurrence probabilities of *s*_1_ and *s*_2_ are unknown but *x*_1_ +*x*_2_ = 0.8 (0.8 = 1–0.2). Besides, we do not consider the situation that there is only one unknown occurrence probability such as *L*_7_(*p*) = {*s*_0_(0.2), *s*_3_(*x*_3_)} because it can be attained by $1-\sum_{k=1}^{\#L(x)}x^{\left(k\right)}$ (*x*^(*k*)^ represents the known probability) [27].

To distinguish between incomplete PLTSs and complete PLTSs, Wang et al. [27] introduced the concept of complete PLTSs (CPLTSs).

**Definition 7** [27] Suppose that $\bar{L}(x)=\left\{{\bar{L}}^{\left(k\right)}\left(x^{\left(k\right)}\right)|k=1,2,\ldots ,\#\bar{L}(x)\right\}$ is an InPLTS, when all the LTs ($\bar{L}(x)$) and their occurrence probabilities (*x*^(*k*)^) are complete and known, i.e., $\sum_{k=1}^{\#\bar{L}(x)}x^{\left(k\right)}=1$, the InPLTS is called complete PLTS(CPLTS).

In the process of normalization, the purpose is to transform the InPLTS into CPLTS so the CPLTS can be regarded as the normalized PLTS. Moreover, in this paper, we do not consider the second task of normalization [5] because the numbers of LTs between two PLTSs can be processed straightforwardly to be same, i.e., #*L*_1_(*p*) = #*L*_2_(*p*).

### 2.3 Entropy measure of PLTS

Entropy is an useful tool to represent the uncertainty of PLTS because the PLTS contains various LTs and occurrence probabilities. Liu et al. [33] developed some methods of entropy measure of PLTS and we review them in the following.

**Definition 8** [33] Suppose that *L*(*p*) = {*L*^(*k*)^(*p*^(*k*)^) | *k* = 1, 2, …, #*L*(*p*)} is a PLTS, then the fuzzy entropy is denoted as *E*_*F*_. The calculation is given by

$$E_{F}(L(p))=1-\sum_{k=1}^{\#L(p)}p^{\left(k\right)}\left|1-2\beta_{k}\right|,$$ (3)

where *β*_*k*_ = *I*(*s*_*k*_)/*g*, *g* = #*L*(*p*)-1.

**Definition 9** [33] Let *L*(*p*) = {*L*^(*k*)^(*p*^(*k*)^) | *k* = 1, 2, …, #*L*(*p*)} be a PLTS, then the hesitant entropy is denotes as *E*_*H*_. The calculation is given by

$$E_{H}(L(p))=\{\begin{array}{cc} \sum_{k=1}^{\#L(p)}\sum_{l=k+1}^{\#L(p)}4p^{\left(k\right)}p^{\left(l\right)}f\left(r^{kl}\right) & ,\#L(p)\geq 2\\ 0 & ,\#L(p)=1 \end{array},$$ (4)

where *r*_*kl*_ = |*β*_*k*_−*β*_*l*_|, $f\left(r_{kl}\right)=r_{kl}^{q}$.

**Remark 3** For the entropy measure of PLTS, Liu et al. [33] proposed six approaches and six formulas to compute the fuzzy entropy and hesitant entropy, respectively. Among them, we choose the relatively simple ones, i.e., Eqs (3) and (4), because the focus in this paper is to explore the entropy change before and after the normalization of PLTS. In addition, for the convenience of calculation below, the value of *q* is set 1 in Eq (4).

## 3. Novel normalization method of PLTS based on minimum entropy change

The normalization of PLTS is an important process in decision making and some methods are stated in the section Introduction. In this section, based on the idea of minimum entropy change, we propose a novel method to normalize the InPLTS, in which the original uncertainty information of InPLTS can be retained to the greatest extent.

### 3.1 Entropy measure of InPLTS

Motivated by fuzzy entropy and hesitant entropy of PLTS [33], the new concepts of InPLTS fuzzy entropy and InPLTS hesitsnt entropy are developed and we design the formulas of entropy measure according to Eqs (3) and (4).

**Definition 10** Let $L(x)=\left\{L^{\left(k\right)}\left(x^{\left(k\right)}\right)|L^{\left(k\right)}\in S,x^{\left(k\right)}\geq 0,k=1,2,\ldots ,\#L(x),\sum_{k=1}^{\#L(x)}x^{\left(k\right)}\leq 1\right\}$ be an InPLTS, then the fuzzy entropy of InPLTS is denoted as *E*_*F*_(*L*(*x*)). The calculation formula is given by

$$E_{F}(L(x))=1-\sum_{k=1}^{\#L(x)}x^{\left(k\right)}\left|1-2\beta_{k}\right|,$$ (5)

**Theorem 1** For any two InPLTSs *L*_*a*_(*x*) and *L*_*b*_(*x*), if $L_{a}^{\left(k\right)}=L_{b}^{\left(k\right)}$, $x_{a}^{\left(k\right)}=x_{b}^{\left(k\right)}$, $L_{a}^{\left(k\right)}\left(x^{\left(k\right)}\right)$ and $L_{b}^{\left(k\right)}\left(x^{\left(k\right)}\right)$ belong to the known elements, then *E*_*F*_(*L*_*a*_(*x*)) = *E*_*F*_(*L*_*b*_(*x*)).

**Proof** Let *L*_*a*_(*x*) and *L*_*b*_(*x*) be two InPLTSs.

$$\begin{array}{cc} E_{F}\left(L_{a}(x)\right) & =1-\left[x_{a}^{1}\left|1-2\alpha_{a}^{1}\right|+x_{a}^{2}\left|1-2\alpha_{a}^{2}\right|+\ldots +x_{a}^{\left(k\right)}\left|1-2\alpha_{a}^{\left(k\right)}\right|\right]\\ & =1-\left[x_{a}^{1}\left|1-2\times \frac{I\left(L_{a}^{1}\right)}{g}\right|+x_{a}^{2}\left|1-2\times \frac{I\left(L_{a}^{2}\right)}{g}\right|+\ldots +x_{a}^{\left(k\right)}\left|1-2\times \frac{I\left(L_{a}^{\left(k\right)}\right)}{g}\right|\right]\\ E_{F}\left(L_{b}(x)\right) & =1-\left[x_{b}^{1}\left|1-2\alpha_{b}^{1}\right|+x_{b}^{2}\left|1-2\alpha_{b}^{2}\right|+\ldots +x_{b}^{\left(k\right)}\left|1-2\alpha_{b}^{\left(k\right)}\right|\right]\\ & =1-\left[x_{b}^{1}\left|1-2\times \frac{I\left(L_{b}^{1}\right)}{g}\right|+x_{b}^{2}\left|1-2\times \frac{I\left(L_{b}^{2}\right)}{g}\right|+\ldots +x_{b}^{\left(k\right)}\left|1-2\times \frac{I\left(L_{b}^{\left(k\right)}\right)}{g}\right|\right] \end{array},$$

where $L_{a}^{\left(k\right)}=L_{b}^{\left(k\right)}$, $x_{a}^{\left(k\right)}=x_{b}^{\left(k\right)}$, $L_{a}^{\left(k\right)}\left(x^{\left(k\right)}\right)$ and $L_{b}^{\left(k\right)}\left(x^{\left(k\right)}\right)$ belong to the known elements. Thus, *E*_*F*_(*L*_*a*_(*x*)) = *E*_*F*_(*L*_*b*_(*x*)).

**Example 1** Suppose that *S* = {*s*_0_, *s*_1_, *s*_2_, *s*_3_, *s*_4_} is a LTS, *L*_1_(*x*) = {*s*_0_(0.3), *s*_1_(0.4), *s*_2_(*x*_2_), *s*_3_(*x*_3_)}, *L*_2_(*x*) = {*s*_0_(0.3), *s*_1_(0.4)}, *s*_α_∈*S*. Because the probabilities of *s*_2_ and *s*_3_ are unknown, the *x*_2_ and *x*_3_ are not considered when computing the fuzzy entropy of InPLTS. Then,

$$\begin{array}{cc} E_{F}\left(L_{1}(x)\right) & =1-\sum_{k=1}^{\#L(x)}x^{\left(k\right)}\left|1-2\beta_{k}\right|\\ & =1-\left[0.3\left|1-2\times \frac{0}{5}\right|+0.4\left|1-2\times \frac{1}{5}\right|\right]\\ & =0.46 \end{array}$$

Similarly, *E*_*F*_(*L*_2_(*x*)) = 0.46. Here, we can also attain the fuzzy entropy of *L*_2_(*x*) by Theorem 1.

Although the final results of *E*_*F*_(*L*_1_(*x*)) and *E*_*F*_(*L*_2_(*x*)) are identical, they represent two different types of InPLTS that *L*_1_(*x*) is the MP-InPLTS and *L*_2_(*x*) is the MLT-InPLTS. We can see that there are four LTs in *L*_1_(*x*) and *s*_2_, *s*_3_ are known elements even though their probabilities are unknown. However, in *L*_2_(*x*), there are only two LTs and the missing probabilities are not able to know certainly which LT to belong to. Thus, the same fuzzy entropy of InPLTSs only means that the two InPLTSs have the identical original uncertainty information.

According to Definition 6 and Eq (3), the fuzzy entropy of CPLTS is given by

$$E_{F}(\bar{L}(x))=1-\sum_{k=1}^{\#\bar{L}(x)}x^{\left(k\right)}\left|1-2\beta_{k}\right|,$$ (6)

Where $\bar{L}(x)$ is a CPLTS, $\sum_{i=1}^{\#\bar{L}(x)}x^{\left(k\right)}=1$. For simplify, the fuzzy entropy of CPLTS is denoted as ${\bar{E}}_{F}$.

**Definition 11** Let $L(x)=\left\{L^{\left(k\right)}\left(x^{\left(k\right)}\right)|L^{\left(k\right)}\in S,x^{\left(k\right)}\geq 0,k=1,2,\ldots ,\#L(x),\sum_{k=1}^{\#L(x)}x^{\left(k\right)}\leq 1\right\}$ be an InPLTS, then the hesitant entropy of InPLTS is denoted as *E*_*F*_(*L*(*x*)). The calculation formula is given by

$$E_{H}(L(x))=\{\begin{array}{cc} \sum_{k=1}^{\#L(x)}\sum_{l=k+1}^{\#L(x)}4x^{\left(k\right)}x^{\left(l\right)}f\left(r^{kl}\right) & ,\#L(x)\geq 2\\ 0 & ,\#L(x)=1 \end{array},$$ (7)

where $f\left(r_{kl}\right)=r_{kl}^{q}$, *q* = 1.

**Theorem 2** For any two InPLTSs *L*_*a*_(*x*) and *L*_*b*_(*x*), if $L_{a}^{\left(k\right)}=L_{b}^{\left(k\right)}$, $x_{a}^{\left(k\right)}=x_{b}^{\left(k\right)}$ and $L_{a}^{\left(k\right)}\left(x^{\left(k\right)}\right)$ and $L_{b}^{\left(k\right)}\left(x^{\left(k\right)}\right)$ belong to the known elements, then *E*_*H*_(*L*_*a*_(*x*)) = *E*_*H*_(*L*_*b*_(*x*)).

**Proof** Let *L*_*a*_(*x*) and *L*_*b*_(*x*) be two InPLTSs.

If #*L_a_*(*x*) = #*L_b_*(*x*) = 0, then *E_H_*(*L_a_*(*x*)) = *E_H_*(*L_b_*(*x*)) = 0

If #*L_a_*(*x*) ≥ 2, #*L_b_*(*x*) ≥ 2, then

$$\begin{array}{cc} E_{H}\left(L_{a}(x)\right) & =4x_{a}^{\left(1\right)}x_{a}^{\left(2\right)}f\left(r_{a}^{12}\right)+4x_{a}^{\left(1\right)}x_{a}^{\left(3\right)}f\left(r_{a}^{13}\right)+\cdots 4x_{a}^{\left(1\right)}x_{a}^{\left(k\right)}f\left(r_{a}^{1k}\right)\\ & +4x_{a}^{\left(2\right)}x_{a}^{\left(3\right)}f\left(r_{a}^{23}\right)+4x_{a}^{\left(1\right)}x_{a}^{\left(2\right)}f\left(r_{a}^{12}\right)+\cdots +4x_{a}^{\left(2\right)}x_{a}^{\left(k\right)}f\left(r_{a}^{2k}\right)+\cdots +4x_{a}^{\left(k-1\right)}x_{a}^{\left(k\right)}f\left(r_{a}^{k-1,k}\right)\\ & =4x_{a}^{\left(1\right)}x_{a}^{\left(2\right)}\left|\frac{I\left(L_{a}^{\left(1\right)}\right)}{g}-\frac{I\left(L_{a}^{\left(2\right)}\right)}{g}\right|+4x_{a}^{\left(1\right)}x_{a}^{\left(3\right)}\left|\frac{I\left(L_{a}^{\left(1\right)}\right)}{g}-\frac{I\left(L_{a}^{\left(2\right)}\right)}{g}\right|+\cdots +4x_{a}^{\left(k-1\right)}x_{a}^{\left(k\right)}\left|\frac{I\left(L_{a}^{\left(k-1\right)}\right)}{g}-\frac{I\left(L_{a}^{\left(k\right)}\right)}{g}\right|\\ E_{H}\left(L_{b}(x)\right) & =4x_{b}^{\left(1\right)}x_{b}^{\left(2\right)}f\left(r_{b}^{12}\right)+4x_{b}^{\left(1\right)}x_{b}^{\left(3\right)}f\left(r_{b}^{13}\right)+\cdots +4x_{b}^{\left(1\right)}x_{b}^{\left(k\right)}f\left(r_{b}^{1k}\right)\\ & +4x_{b}^{\left(2\right)}x_{b}^{\left(3\right)}f\left(r_{b}^{23}\right)+4x_{b}^{\left(2\right)}x_{b}^{\left(4\right)}f\left(r_{b}^{24}\right)+\cdots +4x_{b}^{\left(2\right)}x_{b}^{\left(k\right)}f\left(r_{b}^{2k}\right)+\cdots +4x_{b}^{\left(k-1\right)}x_{b}^{\left(k\right)}f\left(r_{b}^{k-1,k}\right)\\ & =4x_{b}^{\left(1\right)}x_{b}^{\left(2\right)}\left|\frac{I\left(L_{b}^{\left(1\right)}\right)}{g}-\frac{I\left(L_{b}^{\left(2\right)}\right)}{g}\right|+4x_{b}^{\left(1\right)}x_{b}^{\left(3\right)}\left|\frac{I\left(L_{b}^{\left(1\right)}\right)}{g}-\frac{I\left(L_{b}^{\left(3\right)}\right)}{g}\right|+\cdots +4x_{b}^{\left(k-1\right)}x_{b}^{\left(k\right)}\left|\frac{I\left(L_{b}^{\left(k-1\right)}\right)}{g}-\frac{I\left(L_{b}^{\left(k\right)}\right)}{g}\right| \end{array},$$

where $L_{a}^{\left(k\right)}=L_{b}^{\left(k\right)}$, $x_{a}^{\left(k\right)}=x_{b}^{\left(k\right)}$, $L_{a}^{\left(k\right)}\left(x^{\left(k\right)}\right)$ and $L_{b}^{\left(k\right)}\left(x^{\left(k\right)}\right)$ belong to the known elements. Thus, *E*_*H*_(*L*_*a*_(*x*)) = *E*_*H*_(*L*_*b*_(*x*)).

**Example 2** Following example 1, according to Eq (7), the hesitant entropy of *L*_1_(*x*) and *L*_2_(*x*) are as follows:

$$E_{H}\left(L_{1}(x)\right)=4\times 0.3\times 0.4\times \frac{1}{5}=0.096$$

Similarly, *E*_*H*_(*L*_2_(*x*)) = 0.096.

According to Definition 6 and Eq (3), the fuzzy entropy of CPLTS is given by

$$E_{H}(\bar{L}(x))=\{\begin{array}{cc} \sum_{k=1}^{\#\bar{L}(x)}\sum_{l=k+1}^{\#\bar{L}(x)}4x^{\left(k\right)}x^{\left(l\right)}f\left(r^{kl}\right) & ,\#\bar{L}(x)\geq 2\\ 0 & ,\#\bar{L}(x)=1 \end{array},$$ (8)

where $\bar{L}(x)$ is a CPLTS, $\sum_{k=1}^{\#\bar{L}(x)}x^{\left(k\right)}=1$. For simplify, the hesitant entropy of CPLTS is denoted as ${\bar{E}}_{F}$.

**Remark 4** It should be noted that we propose the calculation formula of InPLTS entropy by using Liu’s method, namely, Eqs (5), (6) and (7). However, the difference between Eqs (5) and (3) is that Eq (5) is used to measure an InPLTS with the unknown probabilities or linguistic terms while the Eq (3) is used to measure a PLTS without missing information. In Eq (5), we see that *x*^(*k*)^ represents both known probabilities and unknown probabilities in an InPLTS. In Eq (3), *p*^(*k*)^ only represents known probabilities of PLTS. And, the difference between Eqs (7) and (4) is the same.

For the fuzzy entropy change, hesitant entropy change and total entropy change of InPLTS, we give the definitions below.

**Definition 12** Let $L(x)=\left\{L^{\left(k\right)}\left(x^{\left(k\right)}\right)|L^{\left(k\right)}\in S,x^{\left(k\right)}\geq 0,k=1,2,\ldots ,\#L(x),\sum_{k=1}^{\#L(x)}x^{\left(k\right)}\leq 1\right\}$ be an InPLTS. *E*_*F*_(*L*(*x*)) is the fuzzy entropy of InPLTS and $E_{H}(\bar{L}(x))$ is the fuzzy entropy of corresponding CPLTS. Then the fuzzy entropy change of InPLTS after normalization is given by

$$\Delta E_{F}(L(x))=\left|E_{F}(\bar{L}(x))-E_{F}(L(x))\right|,$$ (9)

where Δ*E*_*F*_(*L*(*x*)) represents the fuzzy entropy change.

**Definition 13** Let $L(x)=\left\{L^{\left(k\right)}\left(x^{\left(k\right)}\right)|L^{\left(k\right)}\in S,x^{\left(k\right)}\geq 0,k=1,2,\ldots ,\#L(x),\sum_{k=1}^{\#L(x)}x^{\left(k\right)}\leq 1\right\}$ be an InPLTS. *E*_*H*_(*L*(*x*)) is the hesitant entropy of InPLTS and $E_{H}(\bar{L}(x))$ is the hesitant entropy of corresponding CPLTS. Then the hesitant entropy change of InPLTS after normalization is given by

$$\Delta E_{H}(L(x))=\left|E_{H}(\bar{L}(x))-E_{H}(L(x))\right|,$$ (10)

where Δ*E*_*H*_(*L*(*x*)) represents the fuzzy entropy change.

Obviously, it is easy to know that the total entropy change of an InPLTS can be derived by Eqs (9) and (10), which is shown below:

$$\Delta E_{T}(L(x))=\Delta E_{F}(L(x))+\Delta E_{H}(L(x)),$$ (11)

where Δ*E*_*T*_(*L*(*x*)) represents the total entropy change.

Wang et al. [27] classified the InPLTS into two types specifically, namely, MP-InPLTS and MLT-InPLTS. Generally, the entropy of InPLTS will be changed in the process of normalization. Based on the idea of minimum entropy change before and after normalization of InPLTS, we propose two optimization models in section 3.2 and section 3.3, which can retain the original information of InPLTS to the greatest extent.

### 3.2 Estimation model of MP-InPLTS

For the MP-InPLTS, it only contains the missing probabilities of known LTs, such as *L*_1_(*x*) = {*s*_0_(0.3), *s*_1_(0.4), *s*_2_(*x*^(2)^), *s*_3_(*x*^(3)^)}. Thus, we only need to consider how to estimate the missing probabilities. And the situation that there only a missing probability in MP-InPLTS is excepted, e.g., *L*(*x*) = {*s*_1_(0.5), *s*_2_(*x*^(2)^)}.

Firstly, calculating the change of fuzzy entropy Δ*E*_*F*_ according to Eq (9),

$$\Delta E_{F}(L(x))=\left|E_{F}(\bar{L}(x))-E_{F}(L(x))\right|=\left|\sum_{k=1}^{\#L(x)}x^{\left(k\right)}\right|1-2\beta^{\left(k\right)}\left|-\sum_{i=1}^{\#\bar{L}(x)}x^{\left(k\right)}\right|1-2\beta^{\left(k\right)}||$$ (12)

Secondly, computing the change of hesitant entropy Δ*E*_*H*_, according to Eq (10),

$$\begin{array}{c} \Delta E_{H}(L(x))=\left|E_{H}(\bar{L}(x))-E_{H}(L(x))\right|\\ =\left|\sum_{k=1}^{\#\bar{L}(x)}\sum_{l=k+1}^{\#\bar{L}(x)}4x^{\left(k\right)}x^{\left(l\right)}f\left(r_{kl}\right)-\sum_{k=1}^{\#L(x)}\sum_{l=k+1}^{\#L(x)}4x^{\left(k\right)}x^{\left(l\right)}f\left(r_{kl}\right)\right| \end{array}$$ (13)

Finally, the total entropy change of InPLTS can be derived by Eqs (11), (12) and (13).


$$\begin{array}{cc} \Delta E_{T}(L(x)) & =\Delta E_{F}(L(x))+\Delta E_{H}(L(x))\\ & =\left|\sum_{k=1}^{\#L(x)}x^{\left(k\right)}\right|1-2\beta_{k}\left|-\sum_{i=1}^{\#\bar{L}(x)}x^{\left(k\right)}\right|1-2\beta_{k}||\\ & +\left|\sum_{k=1}^{\#\bar{L}(x)}\sum_{l=k+1}^{\#\bar{L}(x)}4x^{\left(k\right)}x^{\left(l\right)}f\left(r_{kl}\right)-\sum_{k=1}^{\#L(x)}\sum_{l=k+1}^{\#L(x)}4x^{\left(k\right)}x^{\left(l\right)}f\left(r_{kl}\right)\right| \end{array}$$ (14)

Thus, we construct the optimization model to estimate the missing probabilities. The main idea is to minimize the total entropy change of InPLTS, i.e., min Δ*E*_*T*_. The model is

$$\begin{array}{c} Min\;\Delta E_{T}=\Delta E_{F}+\Delta E_{H}\\ s.t.\;\{\begin{array}{c} \sum_{k=1}^{\#\bar{L}(x)}x^{\left(k\right)}=1\\ \#\bar{L}(x)=\#L(x)\\ \#x^{\left(k\right)}\geq 2\\ 0<x^{\left(k\right)}<1 \end{array} \end{array}$$ (15)

In model (15), the objective function ensures that the change of entropy is minimum after the normalization of InPLTS. The first constraint is to guarantee the sum of probabilities is complete after the normalization. The second and third constraint ensure that the InPLTS is a MP-InPLTS. The final constrain limits the range of *x*^(*k*)^. The constraints above are given to ensure that there exist feasible solutions.

The number of decision variables in model (15) is more than 1 because #*x*^(*k*)^ ≥ 2. It is easy to solve the model, especially when there are only two decision variables. If there are many decision variables, we can use the software LINGO.

To illustrate clearly the model (15) and its merits, we give an example below and have a comparative analysis with Pang’s method.

**Example 3** Following example 1, Suppose that *S* = {*s*_0_, *s*_1_, *s*_2_, *s*_3_, *s*_4_} is a LTS, *L*_1_(*x*) = {*s*_0_(0.3), *s*_1_(0.4), *s*_2_(*x*^(2)^), *s*_3_(*x*^(3)^)}, *s*_*α*_∈*S*. Obviously, *L*_1_(*x*) belongs to MP-InPLTS because there only exist unknown probabilities *x*^(2)^ and *x*^(3)^.

Firstly, we normalize the *L*_1_(*x*) by Pang’s method that the unknown probabilities are averagely assigned into the known linguistic terms. Thus, the normalized result is ${\bar{L}}_{1}(x)=\left\{s_{0}(0.3),s_{1}(0.4),s_{2}(0.15),s_{3}(0.15)\right\}$. According to Eqs (12)–(14), we calculate the total entropy change of *L*_1_(*x*) and the computation processes are as follows:

$$\Delta E_{F}\left(L_{1}(x)\right)=\left|E_{F}\left({\bar{L}}_{1}(x)\right)-E_{F}\left(L_{1}(x)\right)\right|=0.15,$$

$$\Delta E_{H}\left(L_{1}(x)\right)=\left|E_{H}\left({\bar{L}}_{1}(x)\right)-E_{H}\left(L_{1}(x)\right)\right|=0.51,$$

$$\Delta E_{T}\left(L_{1}(x)\right)=\Delta E_{F}\left(L_{1}(x)\right)+\Delta E_{H}\left(L_{1}(x)\right)=0.66.$$

Thus, the total entropy change of *L*_1_(*x*) by using Pang’s method is o.66. Subsequently, we use the proposed model (15) to normalize the *L*_1_(*x*). After computing by Eqs (12)–(14) and pre-process, the optimization model is given by

$$\{\begin{array}{c} Min\;\Delta E_{T}=x_{2}+2.2x_{3}+x_{2}x_{3}\\ x_{2}+x_{3}=0.3 \end{array}.$$

The feasible solution of the model is *x*^(2)^ = 0.3, *x*^(3)^ = 0 and the normalized result is ${\bar{L}}_{1}(x)=\left\{s_{0}(0.3),s_{1}(0.4),s_{2}(0.3),s_{3}(0)\right\}$. The total entropy change derived by the model (15) is Δ*E*_*T*_(*L*_1_(*x*)) = 0.3. Thus, comparing with Pang’s method, the proposed method can reduce the entropy change of InPLTS after normalization process. In other words, the proposed method can better retain the original uncertainty information of InPLTS. In fact, the entropy change of InPLTS is never considered in previous researches so it is a novel and important idea to normalize the InPLTS from the perspective of entropy change.

### 3.3 Estimation model of MLT-InPLTS

The other type of InPLTS is MLT-PLTS, which is also the most common form. For a MLT-PLTS, there exits unknown linguistic term because of the missing probabilities, i.e., $\sum_{k=1}^{\#L(x)}x^{\left(k\right)}<1$. In reality, the rest probability should belong to any possible LT in PLTS [27]. Moreover, we suppose that the rest probability is only assigned to one additive LT because more additive LTs will lead to inaccuracy and distortion of original information.

To estimate the MLT-PLTS, we propose another optimization model which is shown in model (16) according to Eqs (12) and (13).

$$\begin{array}{c} Min\;\Delta E_{T}=\Delta E_{F}+\Delta E_{H}\\ s.t\;.\left\{ \begin{array}{c} \sum_{k=1}^{\#\bar{L}(x)}x^{\left(k\right)}=1\\ {\dot{s}}_{r}\in S\\ \#\bar{L}(x)=\#L(x)+1\\ 0<x^{\left(k\right)}<1 \end{array}\right., \end{array}$$ (16)

where ${\dot{s}}_{r}$ represents the additive LT, and suppose that the additive LT is a discrete integer. In model (16), the objective function is the same as the one in model (15), which ensures the minimum change of entropy. The second and third constrains guarantee that there exist only one additive LT and it belongs to all the LTS *S*. The first and final constrains are to get the CPLTS with the complete probability.

In model (16), there is only one decision variable ${\dot{s}}_{r}$, so the model (16) is easier than model (15) to so solve. To verify the effectiveness and superiority of the model (16), we also give an example below and have a comparative analysis with Pang’s method.

**Example 4** Following example 1, suppose that *S* = {*s*_0_, *s*_1_, *s*_2_, *s*_3_, *s*_4_} is a LTS, *L*_2_(*x*) = {*s*_0_(0.3), *s*_1_(0.4)}, *s*_α_∈*S*. We can see that *L*_2_(*x*) belongs to MLT-InPLTS.

Firstly, according to the proposed method, the normalized form of *L*_2_(*x*) is ${\bar{L}}_{2}(x)=\left\{s_{0}(0.3),s_{1}(0.4),s_{r}(0.3)\right\}$, where *s*_*r*_ ∈ *S*. According to Eqs (12)–(14), we can get fuzzy entropy change $\Delta E_{F}\left({\bar{L}}_{2}(x)\right)$, hesitant entropy change $\Delta E_{H}\left({\bar{L}}_{2}(x)\right)$ and total entropy change $\Delta E_{T}\left({\bar{L}}_{2}(x)\right)$ of *L*_2_(*x*), which are shown as follows:

$$\Delta E_{F}\left(L_{1}(x)\right)=\left|E_{F}\left({\bar{L}}_{1}(x)\right)-E_{F}\left(L_{1}(x)\right)\right|=-0.3\times |1-0.5\text{r}|,$$

$$\Delta E_{H}\left(L_{2}(x)\right)=\left|E_{H}\left({\bar{L}}_{2}(x)\right)-E_{H}\left(L_{2}(x)\right)\right|=0.09r+0.12\times |1-r|,$$

$$\Delta E_{T}\left(L_{2}(x)\right)=\Delta E_{F}\left(L_{2}(x)\right)+\Delta E_{H}\left(L_{2}(x)\right)=0.09r+0.12\times |1-r|-0.3\times |1-0.5\text{r}|.$$

Then, by using the model (16), we construct an optimization model,

$$\begin{array}{c} Min\;\Delta E_{T}=0.09r+0.12\times |1-r|+0.3\times |1-0.5r|\\ s.t.\;\left\{ \begin{array}{c} \sum_{k=1}^{\#\bar{L}(x)}x^{\left(k\right)}=1\\ {\dot{s}}_{r}\in S\\ \#\bar{L}(x)=\#L(x)+1\\ 0<x^{\left(k\right)}<1 \end{array}\right. \end{array}$$

The optimal solution is *r* = 1 and ${\bar{L}}_{2}(x)=\left\{s_{0}(0.3),s_{1}(0.4),s_{1}(0.3)\right\}$, namely, ${\bar{L}}_{2}(x)=\left\{s_{0}(0.3),s_{1}(0.7)\right\}$. When *r* = 1, the total entropy change $\Delta E_{T}\left({\bar{L}}_{2}(x)\right)$ of *L*_2_(*x*) is 0.24.

Subsequently, we use Pang’s method to normalize *L*_2_(*x*) and compute the total entropy change. According to the idea of Pang’s method, the normalized form of *L*_2_(*x*) is ${\bar{L}}_{2}(x)=\{s_{0}(0.43),s_{1}(0.57\}$. By using Eqs (12)–(14), the entropy change of *L*_2_(*x*) can be derived as follows:

$$\Delta E_{F}\left(L_{1}(x)\right)=\left|E_{F}\left({\bar{L}}_{1}(x)\right)-E_{F}\left(L_{1}(x)\right)\right|=0.215,$$

$$\Delta E_{H}\left(L_{2}(x)\right)=\left|E_{H}\left({\bar{L}}_{2}(x)\right)-E_{H}\left(L_{2}(x)\right)\right|=0.125,$$

$$\Delta E_{T}\left(L_{2}(x)\right)=\Delta E_{F}\left(L_{2}(x)\right)+\Delta E_{H}\left(L_{2}(x)\right)=0.340.$$

Obviously, the total entropy change of *L*_2_(*x*) derived by the model (16) is less than the result derived by Pang’s method. Thus, when processing the normalization of MLT-InPLTS, the model (16) can reduce the entropy change to the greatest extent. In other words, it can retain the original uncertainty information of InPLTS to the greatest extent by using the proposed method.

## 4. Model application in multiple attribute group decision making

Based on model (15) and (16), a decision model is constructed to solve the MAGDM problem, which mainly conducts to normalize the incomplete PLTSs of experts. In a MAGDM problem, some mathematical symbols are as follows:

the set of alternatives: *X* = {*x*_1_, *x*_2_, …, *x*_*m*_};

the set of attributes: *C* = {*c*_1_, *c*_2_, …, *c*_*n*_};

the weight vector of attributes: *W* = (*w*_1_, *w*_2_, …, *w*_*n*_)^*T*^;

the set of experts: *E* = {*e*_1_, *e*_2_, …, *e*_*p*_};

the weight vector of experts:*ω* = (*ω*_1_, *ω*_2_, …, *ω*_*p*_)^*T*^.

In our research, suppose that the attribute weights and expert weights are known in advance. There are three steps in the model of MAGDM as follows.

**Step 1**. Estimate the incomplete evaluation information in PLTS. Experts provide the evaluation information over alternatives associated to attributes by the PLTS. The evaluation information from every expert are described as a decision matrix:

$$D^{k}=\left[\begin{array}{cccc} L_{11}^{k} & L_{12}^{k} & \ldots & L_{1n}^{k}\\ L_{21}^{k} & L_{22}^{k} & \ldots & L_{2n}^{k}\\ \ldots & \ldots & \ldots & \ldots \\ L_{m1}^{k} & L_{m1}^{k} & \ldots & L_{mn}^{k} \end{array}\right]$$

Where *D*^*k*^ represents the decision matrix of expert *e*_*k*_, *k* = 1, 2, …, *m*, and $L_{ij}^{k}$ denotes the probability linguistic information of expert *e*_*k*_ over alternative *i* associated to attribute *j* in the form of PLTSs. Due to limited time or the lack of knowledge, experts may provide incomplete information in the form of InPLTSs. We need find out which type the InPLTS belongs to, MP-InPLTS or MLT-InPLTS. If it is a MP-InPLTS, we use the model (15) to estimate the missing information, and otherwise, using the model (16) to estimate MLT-InPLTS.

**Step 2**. Integrate the evaluation information of experts. By step 1, we attain the normalized PLTSs (CPLTSs). In this step, the aggregation operator PLWA (Eq (2)) is utilized to integrate the expert’s decision matrices. Subsequently, the decision matrices are integrated into the decision vector *D*^*T*^ = (*L*_1_, *L*_2_, …, *L*_m_)^*T*^ where *L*_*i*_ represents the collective evaluation over alternative *i*.

**Step 3**. Rank all alternatives and attain the optimal one. The collective evaluation is described by PLTS so we need compare the PLTSs in *D*^*T*^. To conduct that, according to Eq (1), we compute the expected values of PLTSs. Then, the ranking results will be achieved through the comparison of expected values of PLTSs and attained the optimal alternative eventually.

**Fig 1:**
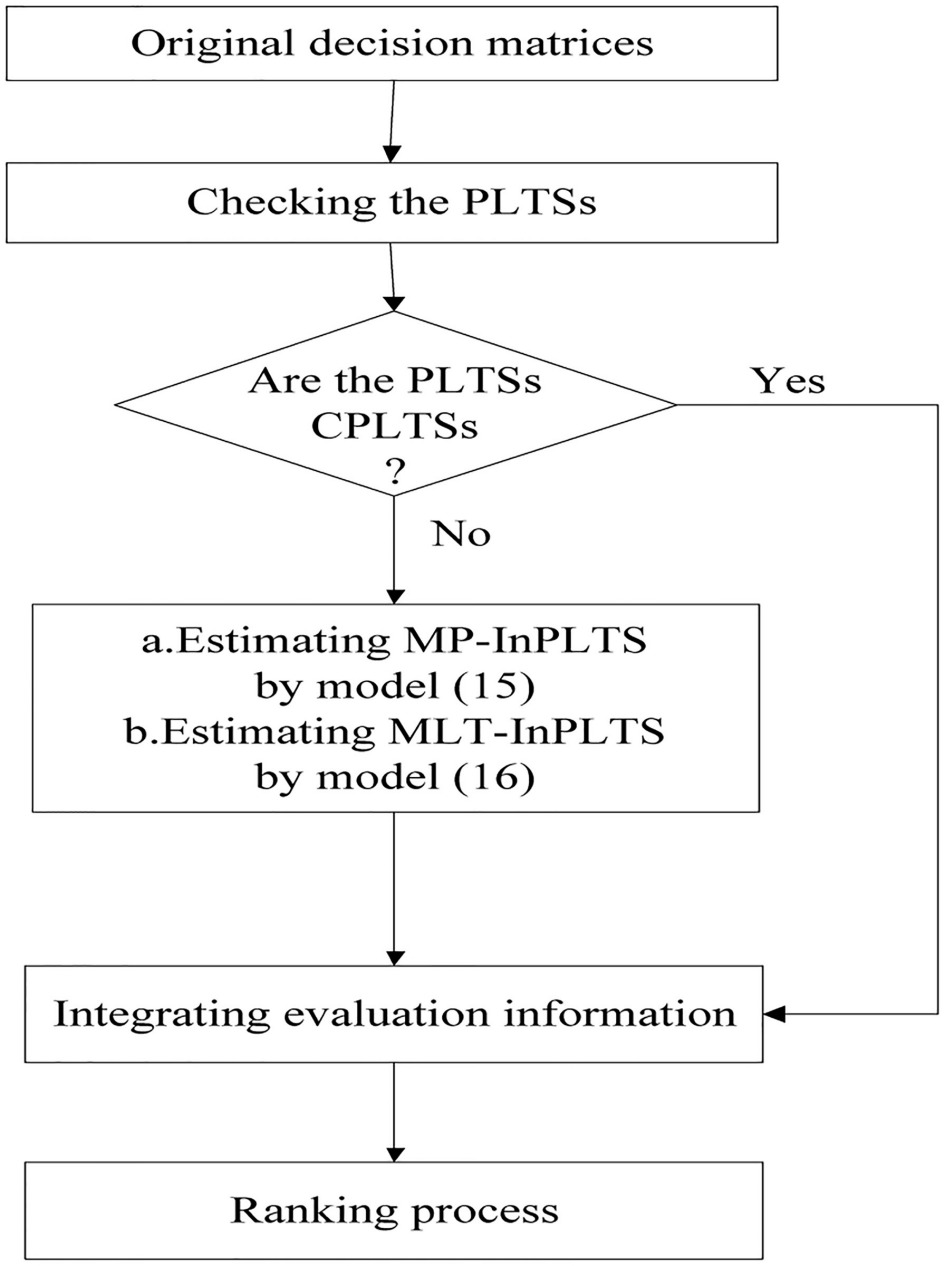
Flowchart of decision making.

In order to intuitively understand the overall decision frame, the flow chart of decision making is given in Fig 1.

## 5. Case study

Tourism is one of the important industries of social and economic development, in which scenic spots are the key components of tourism development. It is not only a special tourism commodity, but also an important place to carry out various tourism activities. Among all scenic spots, the ones with high stars tend to attract more tourists. Thus, star rating is very important for the development of scenic spots, especially for the rating of five-star. In China, the document regulations related to star rating of scenic spots have been issued, in which three important rating indicators are listed as follows:

1. Service quality and environmental quality;
2. Landscape quality;
3. Tourist opinions.

In this way, the rating of five-star can be regarded as a MAGDM problem, in which one optimal scenic spot will be selected as the five-star scenery from all alternatives. Now, suppose that five experts from the tourism field are invited to participate the decision making. Five scenic spots to be selected are Ritan Park, Temple of Heaven Park, Badaling Great Wall, Beijing Olympic Park, and the Summer Palace. Through the evaluations of experts, one of them will become the five-star scenery. However, due to the limited knowledge and experiences, experts can hardly give explicit evaluation information. Therefore, to well depict experts’ preferences, DMs are advised to utilized PLTS as the evaluation expression model. Before processing the MAGDM problem, some symbols are defined mathematically as follows:

1. The five scenic spots are denoted as five alternatives *X* = {*x*_1_, *x*_2_, *x*_3_, *x*_4_, *x*_5_}, where *x*_1_ = ‘Ritan Park’, *x*_2_ = ‘Temple of Heaven Park’ and *x*_3_ = ‘Badaling Great Wall’, *x*_4_ = ‘Beijing Olympic Park’, *x*_5_ = ‘the Summer Palace’.
2. The three indicators are regarded as three attributes *C* = {*c*_1_, *c*_2_, *c*_3_}, where *c*_1_ = ‘Service quality and environmental quality’, *c*_2_ = ‘Landscape quality’ and *c*_3_ = ‘Tourist opinions’.
3. Considering that *c*_1_ is the most important attribute, its attribute weight is the biggest. Thus, the corresponding weight vector of attributes is preset as *W* = (0.5, 0.3, 0.2)^*T*^;
4. The five experts are denoted as *E* = {*e*_1_, *e*_2_, *e*_3_, *e*_4_, *e*_5_} and the weight vector of experts is *ω* = (0.3, 0.2, 0.2, 0.2, 0.1)^*T*^.

Experts use the linguistic term set LTS with five granularity, namely, *S* = {*s*_*α*_|*α* = 0, 1, 2, 3, 4}, where *s*_0_ = ‘exactly bad’, *s*_1_ = ‘bad’, *s*_2_ = ‘indifferent’, *s*_3_ = ‘good’, *s*_4_ = ‘exactly good’. The decision matrices provided by experts are given in Tables 1–5 respectively.

**Table 1:** The decision matrix of *e*_1_.

*e*_1_	*c*_1_	*c*_2_	*c*_3_
*x*_1_	{*s*_0_(0.2), *s*_1_(0.8)}	{*s*_1_(0.3), *s*_2_(0.5)}	{*s*_2_(1)}
*x*_2_	{*s*_1_(0.4), *s*_2_(*x*_2_), *s*_3_(*x*_3_)}	{*s*_3_(1)}	{*s*_2_(0.3), *s*_3_(0.7)}
*x*_3_	{*s*_2_(1)}	{*s*_2_(0.5), *s*_3_(0.5)}	{*s*_1_(0.4), *s*_2_(0.6)}
*x*_4_	{*s*_1_(0.6), *s*_2_(0.4)}	{*s*_0_(1)}	{*s*_1_(1)}
*x*_5_	{*s*_0_(0.3), *s*_2_(0.7)}	{*s*_2_(1)}	{*s*_1_(0.5), *s*_2_(0.5)}

**Table 2:** The decision matrix of *e*_2_.

*e*_2_	*c*_1_	*c*_2_	*c*_3_
*x*_1_	{*s*_0_(0.5), *s*_1_(0.5)}	{*s*_1_(0.6), *s*_2_(0.4)}	{*s*_1_(0.7), *s*_2_(0.3)}
*x*_2_	{*s*_3_(1)}	{*s*_2_(1)}	{*s*_2_(0.4), *s*_3_(0.6)}
*x*_3_	{*s*_2_(1)}	{*s*_2_(0.5), *s*_3_(0.5)}	{*s*_2_(1)}
*x*_4_	{*s*_1_(0.7), *s*_2_(0.3)}	{*s*_1_(0.4), *s*_2_(0.6)}	{*s*_1_(1)}
*x*_5_	{*s*_2_(1)}	{*s*_1_(1)}	{*s*_2_(0.5), *s*_3_(0.5)}

**Table 3:** The decision matrix of *e*_3_.

*e*_3_	*c*_1_	*c*_2_	*c*_3_
*x*_1_	{*s*_1_(1)}	{*s*_1_(0.5), *s*_2_(0.5)}	{*s*_0_(0.5), *s*_1_(*x*_1_), *s*_3_(*x*_3_)}
*x*_2_	{*s*_2_(0.4), *s*_3_(0.6)}	{*s*_2_(1)}	{*s*_2_(0.5), *s*_3_(0.5)}
*x*_3_	{*s*_3_(1)}	{*s*_2_(0.5), *s*_3_(0.4)}	{*s*_2_(1)}
*x*_4_	{*s*_2_(0.5), *s*_3_(0.5)}	{*s*_2_(1)}	{*s*_2_(0.5), *s*_3_(0.5)}
*x*_5_	{*s*_1_(0.4), *s*_2_(0.6)}	{*s*_1_(1)}	{*s*_0_(1)}

**Table 4:** The decision matrix of *e*_4_.

*e*_4_	*c*_1_	*c*_2_	*c*_3_
*x*_1_	{*s*_3_(1)}	{*s*_2_(1)}	{*s*_0_(0.5), *s*_1_(0.5)}
*x*_2_	{*s*_1_(0.5), *s*_2_(0.5)}	{*s*_3_(0.7), *s*_4_(0.3)}	{*s*_2_(0.3), *s*_3_(0.7)}
*x*_3_	{*s*_2_(0.2), *s*_3_(0.8)}	{*s*_0_(1)}	{*s*_2_(1)}
*x*_4_	{*s*_2_(1)}	{*s*_2_(0.5), *s*_3_(0.5)}	{*s*_1_(0.5), *s*_2_(0.5)}
*x*_5_	{*s*_0_(1)}	{*s*_3_(1)}	{*s*_1_(1)}

**Table 5:** The decision matrix of *e*_5_.

*e*_5_	*c*_1_	*c*_2_	*c*_3_
*x*_1_	{*s*_2_(1)}	{*s*_2_(0.7), *s*_3_(0.3)}	{*s*_1_(1)}
*x*_2_	{*s*_1_(0.5), *s*_2_(0.5)}	{*s*_3_(1)}	{*s*_2_(0.5), *s*_3_(0.5)}
*x*_3_	{*s*_3_(1)}	{*s*_0_(0.3), *s*_1_(0.7)}	{*s*_1_(0.3), *s*_2_(0.7)}
*x*_4_	{*s*_2_(1)}	{*s*_2_(1)}	{*s*_3_(1)}
*x*_5_	{*s*_3_(0.7), *s*_4_(0.3)}	{*s*_0_(1)}	{*s*_2_(1)}

According to the proposed MAGDM model in section 4, we solve the decision problem and the steps are as follows:

**Step 1**. Find out the PLTS with missing information in decision matrices, namely, the InPLTSs. In Tables 1–5, we can see that $L_{12}^{1}$, $L_{21}^{1}$, $L_{13}^{3}$ and $L_{32}^{3}$ are InPLTSs. The decision matrices of expert *e*_2_, *e*_4_, and *e*_5_ are complete. Evidently, $L_{21}^{1}=\left\{s_{1}(0.4),s_{2}\left(x^{\left(2\right)}\right),s_{3}\left(x^{\left(3\right)}\right)\right\}$ and $L_{13}^{3}=\left\{s_{0}(0.5),s_{1}\left(x^{\left(1\right)}\right),s_{3}\left(x^{\left(3\right)}\right)\right\}$ belong to MP-InPLTS while $L_{12}^{1}=\left\{s_{1}(0.3),s_{2}(0.5)\right\}$ and $L_{32}^{3}=\left\{s_{2}(0.5),s_{3}(0.4)\right\}$ belong to MLT-InPLTS. Thus, we estimate $L_{21}^{1}$ and $L_{13}^{3}$ by using model (15) and estimate $L_{12}^{1}$, $L_{32}^{3}$ by using model (16). The calculation processes are shown below.

Firstly, we use the model (15) to normalize $L_{21}^{1}$ and $L_{13}^{3}$. We take $L_{21}^{1}$ as an example. According to Eqs (12)–(14), $\Delta E_{F}\left(L_{21}^{1}\right)$, $\Delta E_{H}\left(L_{21}^{1}\right)$ and $\Delta E_{T}\left(L_{21}^{1}\right)$ can be derived:

$$\Delta E_{F}\left(L_{21}^{1}\right)=\left|E_{F}\left({\bar{L}}_{21}^{1}\right)-E_{F}\left(L_{21}^{1}\right)\right|=0.5x^{\left(3\right)}$$

$$\Delta E_{H}\left(L_{21}^{1}\right)=\left|E_{H}\left({\bar{L}}_{21}^{1}\right)-E_{H}\left(L_{21}^{1}\right)\right|=0.4x^{\left(2\right)}+0.8x^{\left(3\right)}+x^{\left(2\right)}x^{\left(3\right)}$$

$$\Delta E_{T}\left(L_{21}^{1}\right)=\Delta E_{F}\left(L_{21}^{1}\right)+\Delta E_{T}\left(L_{21}^{1}\right)=0.4x^{\left(2\right)}+1.3x^{\left(3\right)}+x^{\left(2\right)}x^{\left(3\right)}$$

By using the model (15), we construct the optimal model:

$$\{\begin{array}{c} \text{min}\;\Delta E_{T}\left(L_{21}^{1}\right)=0.4x^{\left(2\right)}+1.3x^{\left(3\right)}+x^{\left(2\right)}x^{\left(3\right)}\\ x^{\left(2\right)}+x^{\left(3\right)}=0.6 \end{array}$$

The feasible solution is *x*^(2)^ = 0.6 and *x*^(3)^ = 0. Thus, ${\bar{L}}_{21}^{1}=\left\{s_{1}(0.4),s_{2}(0.6),s_{3}(0)\right\}$, namely, ${\bar{L}}_{21}^{1}=\left\{s_{1}(0.4),s_{2}(0.6)\right\}$. Subsequently, the normalized result of $L_{32}^{3}$ can be also attained, ${\bar{L}}_{13}^{3}=\left\{s_{0}(0.5),s_{1}(0.5)\right\}$.

Then, according to the model (16), we can get the normalized forms of $L_{12}^{1}$ and $L_{32}^{3}$. Take $L_{12}^{1}$ as an example. Suppose that the normalized form of $L_{12}^{1}$ is $L_{12}^{1}=\left\{s_{1}(0.3),s_{2}(0.5),s_{r}(0.2)\right\}$, where *s*_*r*_ ∈ *S*. By using Eqs (12)–(14), $\Delta E_{F}\left(L_{12}^{1}\right)$, $\Delta E_{H}\left(L_{12}^{1}\right)$ and $\Delta E_{T}\left(L_{12}^{1}\right)$ can be derived:

$$\Delta E_{F}\left(L_{12}^{1}\right)=\left|E_{F}\left({\bar{L}}_{12}^{1}\right)-E_{F}\left(L_{12}^{1}\right)\right|=0.2\times |1-0.5r|$$

$$\Delta E_{H}\left(L_{12}^{1}\right)=\left|E_{H}\left({\bar{L}}_{12}^{1}\right)-E_{H}\left(L_{12}^{1}\right)\right|=0.06\times |1-r|+0.1\times |2-r|$$

$$\Delta E_{T}\left(L_{12}^{1}\right)=\Delta E_{F}\left(L_{12}^{1}\right)+\Delta E_{T}\left(L_{12}^{1}\right)=0.2\times |1-0.5r|+0.06\times |1-r|+0.1\times |2-r|$$

Subsequently, we construct the optimal model as follows:

$$\begin{array}{c} Min\;\Delta E_{T}=0.2\times |1-0.5r|+0.06\times |1-r|+0.1\times |2-r|\\ s.t.\;\left\{ \begin{array}{c} \sum_{k=1}^{\#\bar{L}(x)}x^{\left(k\right)}=1\\ {\dot{s}}_{r}\in S\\ \#\bar{L}(x)=\#L(x)+1\\ 0<x^{\left(k\right)}<1 \end{array}\right. \end{array}$$

The optimal solution of *r* is *r* = 2. Thus, $L_{12}^{1}=\left\{s_{1}(0.3),s_{2}(0.7)\right\}$. And the normalized result of ${\bar{L}}_{32}^{3}$ is ${\bar{L}}_{32}^{3}=\left\{s_{2}(0.5),s_{3}(0.5)\right\}$.

**Step 2**. Integrate the experts’ decision matrices into a collective decision matrix. For the convenience of calculation and aggregation, we transform the PLTSs into the ordered normalized PLTSs according to Definition 3 and the results are shown in Table a in S1 Appendix. The weight vector of experts is *ω* = (0.3, 0.2, 0.2, 0.2, 0.1)^*T*^. By using PLWA operator (Eq (2)), we aggregate the evaluation information of experts and the results are shown in Table 6.

**Table 6:** The collective decision matrix.

	*c*_1_	*c*_2_	*c*_3_
*x*_1_	{*s*_0.69_(0.69), *s*_0_(0.31)}	{*s*_0.96_(0.51), *s*_0.49_(0.49)}	{*s*_1.05_(0.75), *s*_0.3_(0.25)}
*x*_2_	{*s*_2.22_(0.8), *s*_0.28_(0.2)}	{*s*_1.7_(1)}	{*s*_1.83_(0.61), *s*_0.78_(0.39)}
*x*_3_	{*s*_2.2_(1)}	{*s*_1.5_(0.5), *s*_1_(0.5)}	{*s*_1.76_(0.88), *s*_0.12_(0.12)}
*x*_4_	{*s*_1.96_(0.71), *s*_0.65_(0.29)}	{*s*_1.23_(0.68), *s*_2.5_(0.32)}	{*s*_2.15_(0.63), *s*_0.98_(0.37)}
*x*_5_	{*s*_2.34_(0.65), *s*_1.8_(0.35)}	{*s*_1.38_(0.5), *s*_0.98_(0.5)}	{*s*_0.56_(0.85), *s*_0_(0.15)}

Again, we use the PLWA operator to aggregate the collective decision matrix and the aggregation weight is the attribute weight, namely *W* = (0.5, 0.3, 0.2)^*T*^. After aggregating the collective decision matrix, we can get a decision vector, which is shown below:

$$\begin{array}{cc} D^{T}= & (\left\{s_{0.54}(0.65),s_{0.09}(0.35)\right\},\left\{s_{1.62}(0.82),s_{0.09}(0.18)\right\},\left\{s_{1.63}(0.83),s_{0.15}(0.17)\right\},\\ & {\left\{s_{1.47}(0.76),s_{0.12}(0.24)\right\},\left\{s_{1.28}(0.67),s_{0.23}(0.33)\right\})}^{T} \end{array}$$

**Step 3**. Compute the expected values of PLTSs in *D*^*T*^ by Eq (1). The results are *E*(*L*_1_) = 0.3825, *E*(*L*_2_) = 1.3446, *E*(*L*_3_) = 1.3784, *E*(*L*_4_) = 1.146, *E*(*L*_5_) = 0.9335. Then, the ranking of alternatives is *x*_3_ ≻ *x*_2_ ≻ *x*_4_ ≻*x*_5_ ≻*x*_1_.

Therefore, the alternative *x*_3_ is optimal. Namely, according to the evaluations of three experts, Badaling Great Wall is the best scenery and it should be selected as the five-star scenic spot.

## 6. Comparative analysis

In this section, we give the comparative analysis to verify the feasibility and superiority of the proposed method.

### (1) Comparison with Pang’ normalization method of PLTS

In section 5, we see that the incomplete PLTSs are $L_{12}^{1}$, $L_{21}^{1}$, $L_{13}^{3}$ and $L_{32}^{3}$. Among that, $L_{21}^{1}$ and $L_{32}^{3}$ belong to MP-InPLTS while $L_{12}^{1}$ and $L_{32}^{3}$ belong to MLT-InPLTS. The idea of Pang’s normalization method is to averagely assign the rest probabilities to the existing LTs (see Definition 2), namely,

$$\dot{L}(p)=\left\{L^{\left(k\right)}\left({\dot{p}}^{\left(k\right)}\right)|k=1,2,\ldots ,\#L(p)\right\},$$

where ${\dot{p}}^{\left(k\right)}=p^{\left(k\right)}/\sum_{k=1}^{\#L(p)}p^{\left(k\right)}$.

Although Pang’s method seems very simple and straightforward, the existing defects of Pang’s method are evident. Firstly, it is not scientific and accuracy. As we see, the unknown probabilities do not mean that they must belong to the known linguistic terms. In some cases, people may not ensure that the rest probabilities should be assigned to which element. Thus, Pang’s method is rough and inaccuracy. Secondly, Pang’s method can only cope with the MLT-InPLTS while it does not consider the situation of MP-InPLTS. For $L_{21}^{1}$ and $L_{32}^{3}$ in section 5, we can not use Pang’s method to solve. Hence, Pang’s method is limited. Thirdly, the normalized results derived by Pang’s method are counter-intuitive. Especially, if there only exists one element with unknown probability, the probability will be directly normalized as 1. For example, *L*_1_(*p*) = {*s*_0_(0.5)} and *L*_2_(*p*) = {*s*_0_(0.8)}, according to Pang’s method, the normalized results are ${\bar{L}}_{1}(p)=\left\{s_{0}(1)\right\}$ and ${\bar{L}}_{2}(p)=\left\{s_{0}(1)\right\}$. Probabilities of two PLTS are identical after normalization, which are not in accord with our intuition. Fourthly, it will largely change the original uncertainty information of PLTS by using Pang’s method.

Comparing with Pang’s method, the proposed normalization method in this paper is more scientific and accuracy. For the four shortcomings of Pang’s method above, the model (15) and model (16) can overcome.

### (2) Comparison with Wang’s normalization method of PLTS

Wang et al. [27] proposed a two-stage process to estimate the missing information for incomplete probabilistic linguistic preference relations(InPLPRs). In stage 1, the programming model is constructed based on maximum preference consistency. To ensure that there exist feasible solutions, in stage 2, it develops one optimization model based on the minimum uncertainty (minimum entropy) overall. It is more scientific and accuracy than Pang’s method.

However, the existing defects in Wang’s method should not be ignored. For Wang’s normalization method, it mainly applies to the case that experts use the PLPR to compare any two alternatives and it is based on the individual preference consistency. Thus, its scope of application is relatively small. Moreover, the calculation process is much complicated because it contains many models in two stages and need compute all preference values in decision matrix. At length, although the entropy is considered in Wang’s method, it does not take the entropy change into account. Thus, it can not retain the original uncertainty information of InPLTS by using Wang’s method. The problems can be solved by using the proposed method in this paper.

### (3) Comparison with other normalization methods of PLTS

In addition to the comparison with the above two methods, we analyze other normalization methods and compare. The comparative results are shown in Table 7.

**Table 7:** The comparison with different normalization methods of PLTS.

Literature	Normalization Method	Computational complexity	Scope of application	Additional conditions	Whether considering entropy	Whether considering change of uncertainty information
Literature [5]	Averagely assign	Low	MP-InPLTS	No	No	No
Literature [27]	Optimization models	High	MP-InPLTS and MLT-InPLTS	Probabilistic linguistic preference relations(PLPR); PLPR consistence; group consensus	Yes	No
Literature [23]	Full-set	Low	MP-InPLTS	No	No	No
	Power-set	High	MP-InPLTS	No	No	No
	Envelope-set	Low	MP-InPLTS	No	No	No
Literature [30]	Personalized normalization method	Common	MP-InPLTS	Considering individual consistence and group consensus; personal risk attitude	No	No
This paper	Minimum entropy change	Low	MP-InPLTS and MLT-InPLTS	No	Yes	Yes

Comparing with other normalization methods, there are two main advantages for the normalization method proposed in this paper, which are summarized as follows:

1. The proposed method can remain the original uncertainty information of InPLTSs to the greatest extent based on the idea of minimum entropy change of PLTS.
2. No more additional conditions need to be considered so the method is applied universally. The two types of InPLTSs basically cover all situations that there exist missing information in a PLTS. Thus, the normalization method can be used to solve the GDM with PLPRs, MAGDM and other decision problems.

Besides, in order to compare the ranking results of the proposed MAGDM model, we respectively use the decision methods in literature [5, 27]. It is worth noting that the attribute weight and the expert weight are given so that we do not need to decide them in literature [5, 27]. The final ranking results are shown in Table 8.

**Table 8:** The ranking results from different methods.

Method	Ranking result
Pang’s method [5]	*x*_3_ ≻ *x*_2_ ≻ *x*_5_ ≻ *x*_4_ ≻ *x*_1_
Wang’s method [27]	*x*_4_ ≻ *x*_2_ ≻ *x*_3_ ≻ *x*_5_ ≻ *x*_1_
This paper	*x*_3_ ≻ *x*_2_ ≻ *x*_4_ ≻ *x*_5_ ≻ *x*_1_

The ranking result by Pang’s method and the one by the proposed method in this paper are same except for alternative *x*_4_ and *x*_5_. In Wang’s method, the optimal alternative is *x*_4_, which is different from the result by the proposed method. The reason may be that the results of PLTS normalization between Wang’s method and this paper’s are distinct. Of course, there are possibly other reasons leaded to the result in the process of decision making. Different from Pang’s method and Wang’s method, when processing the InPLTSs, we use the optimization models based on minimum change of PLTS entropy. It can retain the uncertainty information of experts to the greatest extent and the decision results will be more accurate.

## 7. Conclusions

The normalization method of InPLTS is an important research topic of PLTS. However, there exist many limitations in previous methods, which lead to the loss of uncertainty information and the inaccuracy of decision results. Entropy is an useful tool to measure uncertainty information of PLTS so we give the definitions about fuzzy entropy, hesitant entropy of InPLTS with aid of Liu’s method. Considering that the entropy of InPLTS will be changed after the normalization process, based on the idea of minimum entropy change, we propose two optimization models to estimate the missing information of MP-InPLTS and MLT-InPLTS respectively. On the basis of entropy measure, we give some definitions of entropy change. The proposed normalization method can retain the original uncertainty information of InPLTSs to the greatest extent. Moreover, it does not need any other additional conditions so it can be applied to most cases of InPLTSs. To apply this normalization method to the MAGDM problem, we construct a decision model based on the idea of minimum entropy change of InPLTS. According to the case analysis and comparison with other methods, it is more effective and superior to use the proposed method when processing the MAGDM problems with InPLTSs.

However, there are some limitations in this paper. Firstly, we use the simple formulas to define the entropy measure of InPLTSs, which may be rough. Secondly, for the optimization model of MLT-InPLTS, we suppose that the missing LTs are discrete in the LTSs, which may not attain the optimal solution. Finally, in the model of MAGDM, we preset subjectively the experts’ weights and attribute weights, which need to be further derived by some objective methods. Of course, the idea of minimum entropy change in InPLTS is firstly proposed in this paper, so the main work is to apply this idea to the normalization process of InPLTS and solve the MAGDM problems. For the existing defects in the normalization method, we will have a further study in next work. Besides, in future, we will also explore how to use this normalization method to solve reliable InPLTSs and apply it to the environment of large-scale group decision making.

## Supporting information

S1 Appendix (DOCX)
