# Supplementary material for: The research for PLTS normalization method based on minimum entropy change and its application in MAGDM problem
Source: PLoS One. 2022 May 6;17(5):e0268158. doi: 10.1371/journal.pone.0268158 (PMC9075675; doi:10.1371/journal.pone.0268158)
Supplement: S1 Appendix — (DOCX) [file pone.0268158.s001.docx]

**S1 Appendix**

Table a.The decision matrices with normalized PLTSs

| *e*_1_ | *c*_1_ | *c*_2_ | *c*_3_ |
| --- | --- | --- | --- |
| *x*_1_ | {*s*_1_(0.8),*s*_0_(0.2)} | {*s*_2_(0.7),*s*_1_(0.3)} | {*s*_2_(1),*s*_2_(0)} |
| *x*_2_ | {*s*_2_(0.6),*s*_1_(0.4)} | {*s*_3_(1),*s*_3_(0)} | {*s*_3_(0.7),*s*_2_(0.3)} |
| *x*_3_ | {*s*_2_(1),*s*_2_(0)} | {*s*_3_(0.5),*s*_2_(0.5)} | {*s*_2_(0.6),*s*_1_(0.4)} |
| *x*_4_ | {*s*_2_(0.4),*s*_1_(0.6)} | {*s*_0_(1),*s*_0_(0)} | {*s*_1_(1),*s*_1_(0)} |
| *x*_5_ | {*s*_2_(0.7),*s*_0_(0.3)} | {*s*_2_(1),*s*_2_(0)} | {*s*_2_(0.5),*s*_1_(0.5)} |

| *e*_2_ | *c*_1_ | *c*_2_ | *c*_3_ |
| --- | --- | --- | --- |
| *x*_1_ | {*s*_1_(0.5),*s*_0_(0.5)} | {*s*_2_(0.4),*s*_1_(0.6)} | {*s*_1_(0.7),*s*_2_(0.3)} |
| *x*_2_ | {*s*_3_(1),*s*_3_(0)} | {*s*_2_(1),*s*_2_(0)} | {*s*_3_(0.6),*s*_2_(0.4)} |
| *x*_3_ | {*s*_2_(1),*s*_2_(0)} | {*s*_3_(0.5),*s*_2_(0.5)} | {*s*_2_(1),*s*_2_(0)} |
| *x*_4_ | {*s*_1_(0.7),*s*_2_(0.3)} | {*s*_2_(0.6),*s*_1_(0.4)} | {*s*_1_(1),*s*_1_(0)} |
| *x*_5_ | {*s*_2_(1),*s*_2_(0)} | {*s*_1_(1),*s*_1_(0)} | {*s*_3_(0.5),*s*_2_(0.5)} |

| *e*_3_ | *c*_1_ | *c*_2_ | *c*_3_ |
| --- | --- | --- | --- |
| *x*_1_ | {*s*_1_(1),*s*_1_(0)} | {*s*_2_(0.5),*s*_1_(0.5)} | {*s*_1_(0.5),*s*_0_(0.5)} |
| *x*_2_ | {*s*_3_(0.6),*s*_2_(0.4)} | {*s*_2_(1),*s*_2_(0)} | {*s*_3_(0.5),*s*_2_(0.5)} |
| *x*_3_ | {*s*_3_(1),*s*_3_(0)} | {*s*_3_(0.5),*s*_2_(0.5)} | {*s*_2_(1),*s*_2_(0)} |
| *x*_4_ | {*s*_3_(0.5),*s*_2_(0.5)} | {*s*_2_(1),*s*_2_(0)} | {*s*_3_(0.5),*s*_2_(0.5)} |
| *x*_5_ | {*s*_2_(0.6),*s*_1_(0.4)} | {*s*_1_(1),*s*_1_(0)} | {*s*_0_(1),*s*_0_(0)} |

| *e*_4_ | *c*_1_ | *c*_2_ | *c*_3_ |
| --- | --- | --- | --- |
| *x*_1_ | {*s*_3_(1),*s*_3_(0)} | {*s*_2_(1),*s*_2_(0)} | {*s*_1_(0.5),*s*_0_(0.5)} |
| *x*_2_ | {*s*_2_(0.5),*s*_1_(0.5)} | {*s*_3_(0.7),*s*_4_(0.3)} | {*s*_3_(0.7),*s*_2_(0.3)} |
| *x*_3_ | {*s*_3_(0.8),*s*_2_(0.2)} | {*s*_0_(1),*s*_0_(0)} | {*s*_2_(1),*s*_2_(0)} |
| *x*_4_ | {*s*_2_(1),*s*_2_(0)} | {*s*_3_(0.5),*s*_2_(0.5)} | {*s*_2_(0.5),*s*_1_(0.5)} |
| *x*_5_ | {*s*_0_(1),*s*_0_(0)} | {*s*_3_(1),*s*_3_(0)} | {*s*_1_(1),*s*_1_(0)} |

| *e*_5_ | *c*_1_ | *c*_2_ | *c*_3_ |
| --- | --- | --- | --- |
| *x*_1_ | {*s*_2_(1),*s*_2_(0)} | {*s*_2_(0.7),*s*_3_(0.3)} | {*s*_1_(1),*s*_1_(0)} |
| *x*_2_ | {*s*_2_(0.5),*s*_1_(0.5)} | {*s*_3_(1),*s*_3_(0)} | {*s*_3_(0.5),*s*_2_(0.5)} |
| *x*_3_ | {*s*_3_(1),*s*_3_(0)} | {*s*_1_(0.7),*s*_0_(0.3)} | {*s*_2_(0.7),*s*_1_(0.3)} |
| *x*_4_ | {*s*_2_(1),*s*_2_(0)} | {*s*_2_(1),*s*_2_(0)} | {*s*_3_(1),*s*_3_(0)} |
| *x*_5_ | {*s*_3_(0.7),*s*_4_(0.3)} | {*s*_0_(1),*s*_0_(0)} | {*s*_2_(1),*s*_2_(0)} |
